# Molecular Analysis of Forensically Important Blow Flies in Thailand

**DOI:** 10.3390/insects9040159

**Published:** 2018-11-08

**Authors:** Narin Sontigun, Kabkaew L. Sukontason, Jens Amendt, Barbara K. Zajac, Richard Zehner, Kom Sukontason, Theeraphap Chareonviriyaphap, Anchalee Wannasan

**Affiliations:** 1 Department of Parasitology, Faculty of Medicine, Chiang Mai University, Chiang Mai 50200, Thailand; narinsontigun@gmail.com (N.S.); kabkaew.s@cmu.ac.th (K.L.S.); kom.s@cmu.ac.th (K.S.); 2 Institute of Legal Medicine, Forensic Biology/Entomology, Kennedyallee 104, Frankfurt am Main 60596, Germany; amendt@em.uni-frankfurt.de (J.A.); zehner@em.uni-frankfurt.de (R.Z.); 3 Department for Forensic Genetics, Institute of Forensic Medicine and Traffic Medicine, Voßstraße 2, Heidelberg 69115, Germany; barbarakarolina.zajac@med.uni-heidelberg.de; 4 Department of Entomology, Faculty of Agriculture, Kasetsart University, Bangkok 10900, Thailand; faasthc@ku.ac.th

**Keywords:** forensic entomology, molecular identification, *COI*, *COII*, blow flies, Thailand

## Abstract

Blow flies are the first insect group to colonize on a dead body and thus correct species identification is a crucial step in forensic investigations for estimating the minimum postmortem interval, as developmental times are species-specific. Due to the difficulty of traditional morphology-based identification such as the morphological similarity of closely related species and uncovered taxonomic keys for all developmental stages, DNA-based identification has been increasing in interest, especially in high biodiversity areas such as Thailand. In this study, the effectiveness of long mitochondrial cytochrome *c* oxidase subunit I and II (*COI* and *COII*) sequences (1247 and 635 bp, respectively) in identifying 16 species of forensically relevant blow flies in Thailand (*Chrysomya bezziana*, *Chrysomya chani*, *Chrysomya megacephala*, *Chrysomya nigripes*, *Chrysomya pinguis*, *Chrysomya rufifacies*, *Chrysomya thanomthini*, *Chrysomya villeneuvi*, *Lucilia cuprina*, *Lucilia papuensis*, *Lucilia porphyrina*, *Lucilia sinensis*, *Hemipyrellia ligurriens*, *Hemipyrellia pulchra*, *Hypopygiopsis infumata*, and *Hypopygiopsis tumrasvini*) was assessed using distance-based (Kimura two-parameter distances based on Best Match, Best Close Match, and All Species Barcodes criteria) and tree-based (grouping taxa by sequence similarity in the neighbor-joining tree) methods. Analyses of the obtained sequence data demonstrated that *COI* and *COII* genes were effective markers for accurate species identification of the Thai blow flies. This study has not only demonstrated the genetic diversity of Thai blow flies, but also provided a reliable DNA reference database for further use in forensic entomology within the country and other regions where these species exist.

## 1. Introduction

Among necrophagous insects, blow flies (Diptera: Calliphoridae) are the first comers to colonize on a corpse within a few hours after death [1,2,3]. Therefore, the age of developing blow flies on a corpse can be used to estimate a minimum postmortem interval (PMI_min_), which is the window of time between the day when insects first colonized the body and when the corpse is found [4]. Since developmental times of blow flies are species-specific even between closely related species, correct species identification is a crucial step for accurate PMI_min_ estimation [5]. Traditionally, blow flies would be identified using morphology, but the available taxonomic keys do not provide features for all developmental stages (e.g., immature stages), and the lack of important morphological characteristics in partial or damaged specimens makes morphological identification difficult. DNA-based identification has been intensively carried out for forensically important blow flies [6,7,8]. Additionally, the obtained DNA sequences can provide an estimate of evolutionary history and relationships among organisms via phylogenetic analysis [9,10,11]. Regions of mitochondrial DNA (mtDNA) and nuclear DNA (nuDNA) are widely used for molecular-based discrimination between species. However, the advantageous features of mtDNA (i.e., high copy number per cell, high rate of nucleotide substitutions, and haploid maternal inheritance) make them more ideal for species identification [12]. Among mtDNA, the cytochrome *c* oxidase subunit I and II (*COI* and *COII*) genes have been generally used for species discrimination as well as evolutionary relationship studies because they have a high rate of genetic variation [7,8,13,14,15,16,17].

In Thailand, a total of 93 blow fly species belonging to nine subfamilies and 27 genera have been recognized [18]. In particular, genera *Chrysomya* Robineau-Desvoidy, *Lucilia* Robineau-Desvoidy, *Hemipyrellia* Townsend, and *Hypopygiopsis* Townsend are often present in carrion fly surveys in the country [19,20,21,22]. In addition, 10 species were reported from human corpses as forensically important blow fly evidence, i.e., *Chrysomya megacephala* (Fabricius), *Chrysomya rufifacies* (Macquart)*, Chrysomya villeneuvi* Patton, *Chrysomya nigripes* Aubertin, *Chrysomya bezziana* Villeneuve, *Chrysomya chani* Kurahashi, *Chrysomya pinguis* (Walker), *Lucilia cuprina* (Wiedemann), *Lucilia porphyrina* (Walker), and *Hemipyrellia ligurriens* (Wiedemann) [23,24,25]. So far, blow flies in Thailand have been primarily identified based on available taxonomic keys which cannot permit identification of all life stages [18,25,26,27]. Recently, difficulties in morphology-based identification have been solved by using the molecular approach. In 2010, partial sequences of *COI* and *COII* (1324 bp) were used for the species identification of three forensically important blow fly species, *C. megacephala*, *C. rufifacies*, and *L. cuprina*, from field work in six provinces of Thailand [28]. In 2016, the discrimination of 13 species of forensically relevant flies (nine species of blow flies and four species of flesh flies) from northern Thailand was achieved using nuclear *28S* rRNA (~1000 bp) and mitochondrial *COI* (~700 bp) genes [6]. The immature stages of *C. villeneuvi*, *C. pinguis*, and *L. porphyrina* from human corpses in Chiang Mai province were also identified using morphology and *COI* analyses (1247 bp), and the two latter blow flies have subsequently been included as flies of forensic importance in Thailand [24]. Apart from those publications, genetic data related to forensic entomology in Thailand are still scarce. Thus, it is necessary to establish a reliable local DNA reference database for rapid and accurate species identification. Hence, the aim of this study was to generate nearly full lengths of the *COI* and *COII* sequences of 16 forensically important Thai blow fly species and then evaluate the effectiveness of both genes for accurate species identification.

## 2. Materials and Methods

### 2.1. Specimen Collection

The total of 113 adult blow fly specimens used in this study were obtained from one of three ways: field collections, laboratory colony, and myiasis case (Supplementary Table S1). Firstly, 106 blow flies were collected from various field areas in six provinces of Thailand during March 2013 to March 2015 (Supplementary Figure S1). Briefly, the flies were attracted with 300 g of 1-day-tainted beef offal and were caught using either the semi-automatic funnel trap [22] or a sweep net. The specimens from the field collection were sacrificed by (1) spraying them with and then keeping them in 85% ethanol; (2) placing them in each test tube and then keeping in a freezer box; and (3) keeping them alive before transporting them back to the laboratory for sacrificing by freezing at −20 °C for 2 h. All of the specimens were morphologically identified using the taxonomic key of Kurahashi and Bunchu [18] and subsequently preserved in either 85% ethanol or kept as dried specimens until they were used for molecular analysis. Secondly, six specimens of blow flies were obtained from the laboratory colony of the Department of Parasitology, the Faculty of Medicine, Chiang Mai University (Supplementary Table S1). Lastly, a *C. bezziana* maggot that was received from the ocular myiasis of a patient admitted to Maharaj Nakorn Chiang Mai Hospital in October 2014 was reared into an adult (Supplementary Table S1). In addition, two adult house flies, *Musca domestica* Linnaeus, were obtained from the same rearing laboratory and used as an outgroup.

### 2.2. DNA Extraction, Amplification, and Sequencing

Following the manufacturer’s protocol, genomic DNA was extracted from one or two legs of each fly using the Phire Animal Tissue Direct PCR Kit (Thermo Scientific, Waltham, MA, USA). The remaining parts of the fly were kept as voucher specimens at the Fly Research Unit, the Department of Parasitology, the Faculty of Medicine, Chiang Mai University.

DNA amplification was carried out according to the manufacturer’s protocol for using the Phire Animal Tissue Direct PCR Kit (Thermo Scientific, Waltham, MA, USA). Partial *COI* sequences were amplified using the primers TY-J-1460 (5′-TACAATTTATCGCCTAAACTTCAGCC-3′) and C1-N-2800 (5′-CATTTCAAGCTGTGTAAGCATC-3′) [13], while the primers C2-J-3138 (5′-AGAGCCTCTCCTTTAATAGAACA-3′) [29] and TK-N-3775 (5′-GAGACCATTACTTGCTTTCAGTCATCT-3′) [30] were used for amplification of partial *COII* sequences. The cycling conditions consisted of an initial denaturation step at 98 °C for 5 min followed by 40 cycles of denaturation at 98 °C for 5 s, annealing at 61.7 °C (for *COI*) or 45 °C (for *COII*) for 5 s, extension at 72 °C for 30 s, and a final extension at 72 °C for 1 min. The amplified PCR products were electrophoretically separated on 1% agarose gel, stained with RedSafe^TM^ Nucleic Acid Staining Solution (20,000×) (iNtRon BiotechnologySeongnam-si, South Korea), and visualized under UV light. The PCR products were purified using QIAquick^®^ PCR Purification Kit (QIAGEN, Hilden, Germany) according to the manufacturer’s instructions and subsequently sent to First BASE Laboratories Sdn. Bhd. (Selangor, Malaysia) for Sanger sequencing. The purified PCR products were bidirectionally sequenced with the BigDye^®^ Terminator v3.1 Cycle Sequencing Kit (Applied Biosystems, Foster City, CA, USA) by using the same primers as used in the PCR.

### 2.3. DNA Sequence Alignment and Sequence Analysis

For both *COI* and *COII*, the obtained sequences were manually edited and assembled into the complete bidirectional consensus sequences using BioEdit software version 7.0.9.0. [31]. Within the same species, the number of polymorphic sites and haplotypes for both genes (*COI* and *COII*) were evaluated by DnaSP software version 5.10.01 [32]. The genetic variation within (intraspecific) and between (interspecific) species was calculated using the Kimura two-parameter (K2P) model [33] in MEGA6 software [34]. Furthermore, the frequency distribution of the intra- and interspecific K2P distances was plotted for each gene to observe the overlap in genetic variability.

### 2.4. Nucleotide Sequence Accession Numbers

A representative sequence of identical conspecific haplotypes isolated from the specimens collected from the same area and province were deposited in GenBank; 90 *COI* and 67 *COII* representative sequences of blow flies were assigned accession numbers as KR921597-KR921686 and KU556169-KU556235 (Supplementary Tables S2 and S3), respectively. Notably, this study was the first to add *COI* sequences for *L. sinensis* and *H. tumrasvini* and *COII* sequences for *C. chani, C. thanomthini*, *L. sinensis*, and *H. tumrasvini* into GenBank.

### 2.5. DNA Marker Assessments (COI and COII) for Species Identification

#### 2.5.1. Distance-Based Analysis

Distance-based identification of specimens was performed using SpeciesIdentifier software version 1.8 [35] based on the K2P distance. The percentage of correctly identified specimens of each gene was estimated using the Best Match (BM), Best Close Match (BCM) and All Species Barcodes (ASB) criteria as described by Meier et al. [35]. It is important to note that a sequence without a conspecific match in the dataset will be assigned as incorrect identification. Thus, a sequence of *C. bezziana* was duplicated before analysis. This brought the total of analyses to 114 *COI* and 101 *COII* sequences.

#### 2.5.2. Tree-Based Analysis

The neighbor-joining (NJ) tree was used to identify species using the criteria of Hebert et al. [36]. NJ trees of both genes (*COI* and *COII*) were constructed using the K2P model [33] and tested using 1000 bootstrap replications in MEGA6 software [34]. *Musca domestica* was used as an outgroup in all analyses.

## 3. Results

### 3.1. Sequence Analysis

The partial *COI* amplicons (1247 bp) of 113 blow fly specimens were all successfully sequenced (Supplementary Table S1). The alignments of these 113 *COI* blow fly sequences revealed 300 variable sites and 284 parsimony informative sites with no indels. The average A + T content from all blow fly sequences was 69.5% (A = 30.7% and T = 38.8%), while the average G + C content was 30.5% (C = 14.9% and G = 15.6%). When excluding the species represented by a single sequence (*C. bezziana*), the number of polymorphic sites within a species ranged from 1 (*C. thanomthini*) to 31 (*L. cuprina*), and the number of haplotypes within a species ranged from 2 (*C. thanomthini* and *H. tumrasvini*) to 7 (*C. pinguis*) (Table 1 and Supplementary Table S2).

Of 113 blow fly specimens, the partial *COII* amplicons (635 bp) of 110 blow fly specimens were successfully sequenced (Supplementary Table S1). However, 10 sequences of all *H. ligurriens* specimens showed a 30 bp deletion in the multiple alignment. Thus, the sequences of *H. ligurriens* were tested for pseudogenes by protein translation using the invertebrate mitochondrial codes in MEGA6 software [34]. As stop codons appeared after the deletion, it was assumed that nuclear mitochondrial DNA insertions (NUMTs) were amplified instead of the target gene. Therefore, all *H. ligurriens* sequences were omitted from the analysis. The multiple alignment of the 100 blow fly sequences showed 154 variable sites and 148 parsimony informative sites. The average A + T content from all blow fly sequences was 73.3% (A = 33.3% and T = 40.0%), while the average G + C content was 26.7% (C = 14.2% and G = 12.5%). When excluding species represented by a single sequence (*C. bezziana*), the number of polymorphic sites within a species varied from 0 (*C. megacephala* and *C. chani*) to 14 (*L. porphyrina*), and the number of haplotypes within a species varied from 1 (*C. megacephala* and *C. chani*) to 5 (*L. porphyrina*) (Table 2 and Supplementary Table S3).

### 3.2. Genetic Variation

**Figure 1:**
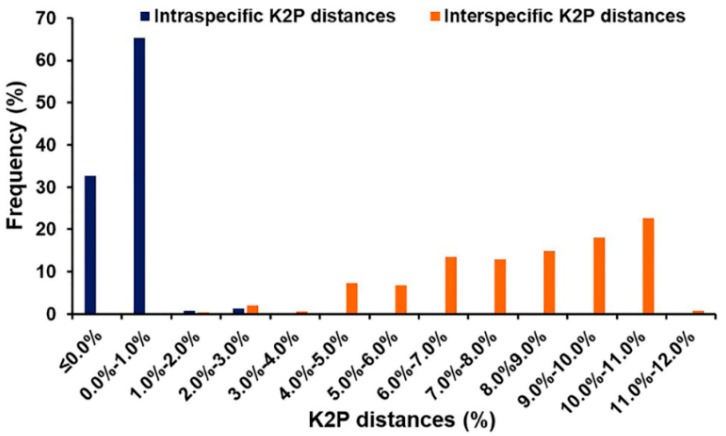
Frequency distribution of intra- and interspecific K2P distances among Thai blow flies based on 1247 bp of *COI* sequences.

Based on 1247 bp of *COI* sequences, the mean intraspecific K2P distances of blow flies ranged from 0.1 to 0.7% (Table 1) while the mean interspecific K2P distances ranged from 1.1% (*H. ligurriens*/*H. pulchra*) to 11.1% (*C. bezziana*/*L. porphyrina*) (Table 3). The mean interspecific K2P distances within Chrysomyinae varied from 2.6% (*C. megacephala*/*C. pinguis*) to 8.1% (*C. bezziana*/*C. rufifacies*) whereas within Luciliinae, it ranged from 1.1% (*H. ligurriens*/*H. pulchra*) to 9.0% (*L. papuensis*/*H. infumata* and *L. porphyrina*/*H. infumata*). Between subfamilies, the mean interspecific K2P distances ranged from 7.9% (*C. chani*/*L. cuprina*) to 11.1% (*C. bezziana*/*L. porphyrina*). However, a small overlap (1.4% from 1.0% to 2.4%) was observed between intra- and interspecific K2P distances (Figure 1).

**Figure 2:**
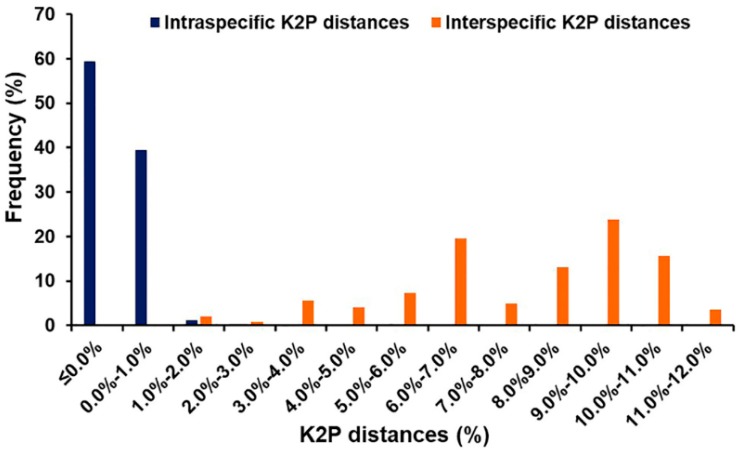
Frequency distribution of intra- and interspecific K2P distances among Thai blow flies based on 635 bp of *COII* sequences.

Based on 635 bp of *COII* sequences, the mean intraspecific K2P distances of blow flies ranged from 0.0 to 0.8% (Table 2), while the mean interspecific K2P distances varied from 1.8% (*C. megacephala*/*C. pinguis*) to 11.6% (*C. nigripes*/*L. papuensis*) (Table 3). Within Chrysomyinae, the divergence varied from 1.8% (*C. megacephala*/*C. pinguis*) to 7.2% (*C. pinguis*/*C. villeneuvi*), whereas the divergence within Luciliinae ranged from 4.2% (*H. pulchra*/*H. infumata*) to 9.9% (*L. papuensis*/*H. infumata*). The mean interspecific K2P distances between subfamilies ranged from 7.0% (*C. chani*/*H. tumrasvini*) to 11.6% (*C. nigripes/L. papuensis*). However, a small overlap (0.1% from 1.8% to 1.9%) was observed between intra- and interspecific K2P distances (Figure 2).

### 3.3. Distance-Based Identification

The percentage of correct identifications for *COI* and *COII* sequences of blow flies was similar (Table 4), revealing very high identification successes for BM (*COI*: 100%; *COII*: 100%), BCM (*COI*: 98.24%; *COII*: 98.01%) and ASB (*COI*: 94.73%; *COII*: 94.05%) criteria. No incorrect identification was observed under the three criteria of both genes (Table 4). However, ambiguous identifications (*COI*: 3.50%; *COII*: 3.96%) were found only when the ASB criterion was used. The BCM and ASB criteria showed 1.75% (*COI*: LC24-DL3, LPO31-1) and 1.98% (*COII*: LPO31-1, LS33-1) of sequences with no matches closer than the calculated threshold (*COI*: 0.72%; *COII*: 0.63%) (Table 4).

### 3.4. Tree-Based Identification

**Figure 3:**
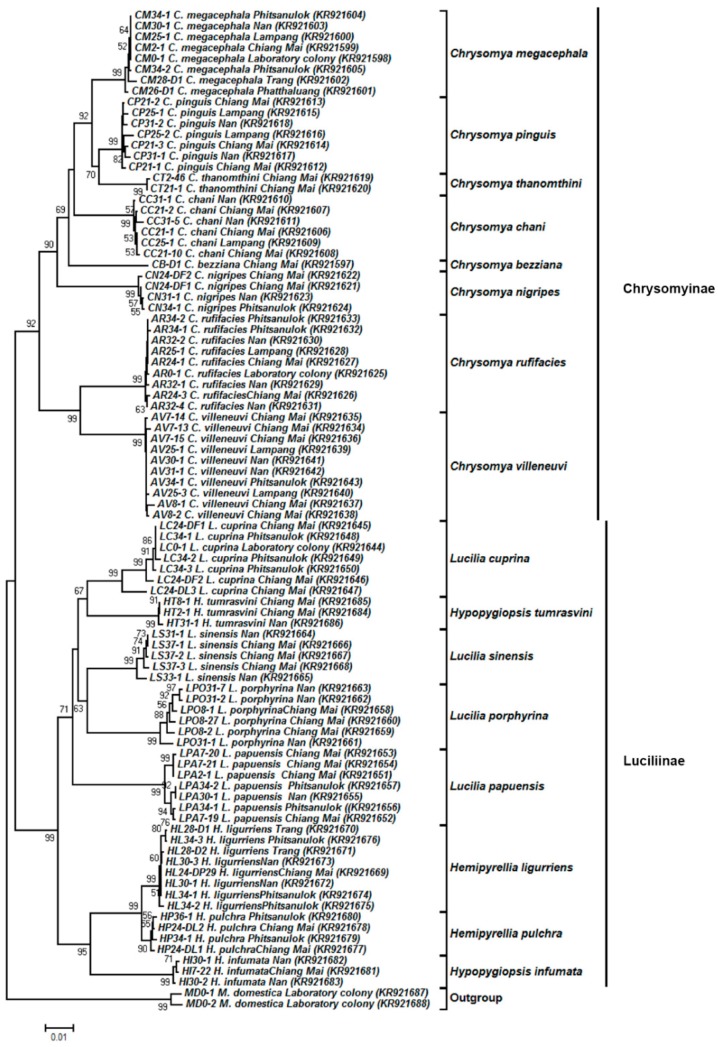
Neighbor-joining tree of *COI* sequences (1247 bp) based on the Kimura two-parameter model. Each specimen label includes the voucher code, species name, collection site, and accession number in parentheses, respectively. Bootstrap values (>50%) are given near the appropriate node. The scale bar 0.01 indicates the evolutionary distance divergence.

**Figure 4:**
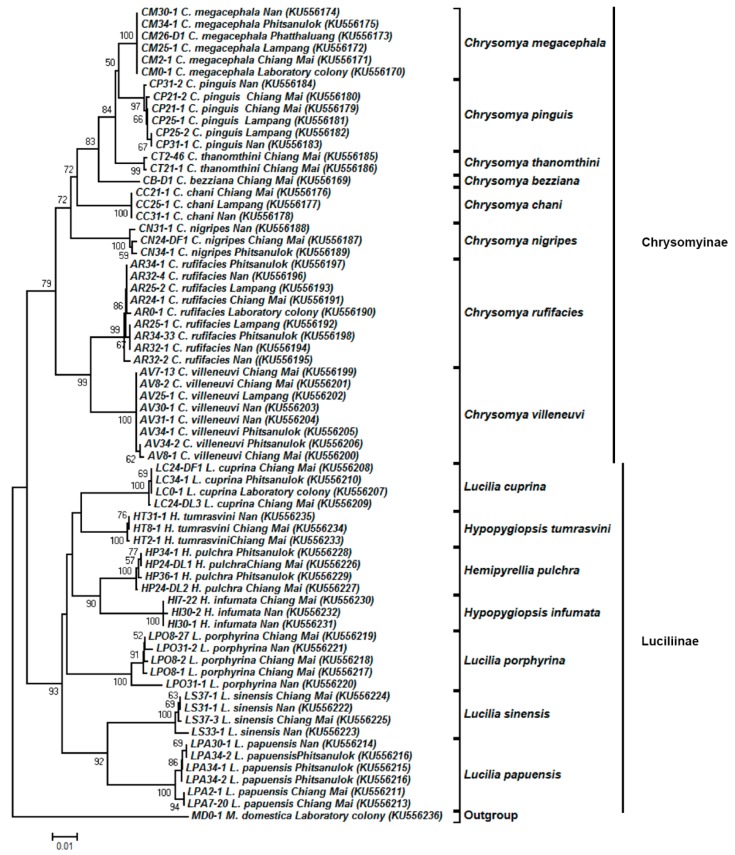
Neighbor-joining tree of *COII* sequences (635 bp) based on the Kimura two-parameter model. Each specimen label includes the voucher code, species name, collection site, and accession number in parentheses, respectively. Bootstrap values (>50%) are given near the appropriate node. The scale bar 0.01 indicates the evolutionary distance divergence.

The NJ trees of both *COI* and *COII* sequences were similar and clearly separated the blow flies from the house fly outgroup (Figure 3 and Figure 4). For 90 representative *COI* sequences (1247 bp), all of 16 blow fly species showed a clear monophyletic cluster with high bootstrap support (90–99%) (Figure 3). The blow flies were divided into two major clades consisting of subfamilies Chrysomyinae and Luciliinae, with bootstrap values of 92% and 99%, respectively. Within Chrysomyinae, the genus *Chrysomya* was separated into two distinct clades; the major clade comprised six *Chrysomya* species (*C. megacephala*, *C. pinguis*, *C. thanomthini*, *C. chani*, *C. bezziana* and *C. nigripes*), while the minor one consisted of *C. rufifacies* and *C. villeneuvi*. Luciliinae was divided into two distinct clades comprising the *Lucilia* clade and the *Hemipyrellia/Hypopygiopsis* clade, both with high bootstrap support (99%). Interestingly, *H. tumrasvini* was placed together with the clade of *Lucilia*, possibly establishing this clade as a sister to *L. cuprina*. 

Regarding 67 representative *COII* sequences (635 bp), 15 blow fly species (excluding the species of *H. ligurriens* of which NUMTs were retrieved) showed the monophyly of each species with high bootstrap support (97–100%) (Figure 4). Two main distinct clades of the blow fly subfamilies, Chrysomyinae and Luciliinae, were also recovered using *COII* with bootstrap values of 79% and 93%, respectively. Tree topology of Chrysomyinae was similar to that of *COI*. Within Luciliinae, the genera *Hemipyrellia* and *Hypopygiopsis* each formed a monophyletic group within the *Lucilia* clade with a low bootstrap value (<50%). Similar to the *COI* tree, *H. tumrasvini* formed a sister clade near *L. cuprina*.

## 4. Discussion

Although molecular-based identification has become widely accepted as an alternative to conventional morphology-based methods for identification in forensic entomology in Thailand, studies examining the genetic variation in targeted regions are scarce. Furthermore, a reference database of sequences from DNA barcode regions commonly used to identify the species of many forensically important flies from Thailand is still needed. Recently, our team assessed the use of partial sequences of nuclear 28*S* rRNA (1000 bp) and mitochondrial *COI* (~700 bp) genes in discriminating some Thai fly species. The study revealed that *COI* appeared to be more conclusive for species discrimination than 28*S* rRNA, with no overlap between the intra- and interspecific distances [6]. In the present study, nearly full lengths of *COI* and *COII* sequences of 16 forensically relevant blow fly species in Thailand were evaluated for their efficacy in accurate species identification.

Although the universal *COII* primers used in this study have been known to work well for other blow flies [14,17,37] and flesh flies [38], the nuclear copies of mtDNA (NUMTs) were amplified instead of the authentic target mtDNA of all Thai *H. ligurriens* specimens. The NUMTs herein were detected by the presence of a premature stop codon, resulting in ambiguity into the COII alignment. Similarly, in a study of mitochondrial genome evolution of *Chrysomya*, most of the sequences of the typical insect *trnQ* gene was missing, and the partially duplicated *trnQ* was noted as a pseudogene [39]. Therefore, it is important to be aware of NUMTs when mtDNA is primarily used for phylogenetic studies. To avoid the co-amplification of NUMTs, several methods have been proposed, such as the use of mtDNA-rich tissue or fresh specimens, amplification of longer fragments, use of specific primers (e.g., taxon-specific primers), improving the PCR conditions, and using reverse transcriptase-coupled PCR (RT-PCR) [40,41,42]. Since NUMTs were only found in *H. ligurriens* when using the universal *COII* primers in this study, the use of specific primers should be considered in further genetic studies of *COII* of Thai *H. ligurriens*.

Intra- and interspecific distances are important for discriminating closely related species, because if the interspecific (congeneric) distances are less than the intraspecific distances, the taxonomic position indicated by the analyzed locus might be generally ambiguous and not be monophyletic [15,43]. However, the overlap between intra- and interspecific distances can occur even though each species is reciprocally monophyletic [43]. In this study, the *COI* data of most species had greater variability than those from the *COII* data. Most intraspecific distances of both *COI* and *COII* genes were less than 1%, with the exception of *L. cuprina* (*COI*: 2.4%) and *L. porphyrina* (*COI*: 1.1%; *COII*: 1.9%), while most interspecific distances were greater than 3%, with the exception of four closely related species pairs (*C. megacephala*/*C. pinguis* (*COI*: 2.6%; *COII*: 1.8%), *C. megacephala*/*C. thanomthini* (*COII*: 2.2%), *C. pinguis*/*C. thanomthini* (*COI*: 2.8%; *COII*: 2.5%), and *H. ligurriens/H. pulchra* (*COI*: 1.1%)). Although some overlap between intra- and interspecific distances was observed in this study, the genetic diversity of *COI* and *COII* genes were sufficient for differentiating among 16 species of Thai blow flies; all species were reciprocally monophyletic on the NJ trees with high bootstrap support (>90%), and identification success based on the three criteria—BM, BCM, and ASB—was very high (>94%), with no incorrect identification. Therefore, *COI* and *COII* genes are effective markers for the identification of forensically important blow fly species in Thailand. Similar findings have been reported in previous studies in the blow flies in other countries [7,8,14,16,17,44,45]. 

According to the study by Meier et al. [35], the three criteria—BM, BCM, and ASB—primarily rely on the sequence pairwise comparison. Therefore, some factors, such as sample size, species diversity, relatedness of species, geographical scale of sampling, and the use of GenBank sequences (which might include some misidentified sequences) in the analyzed dataset can affect the analysis [6,35,43,46,47,48]. Especially in the BCM and ASB criteria, genetic distances are needed for calculating the threshold value, therefore conspecific and congeneric sequences may act as biased data and influence the calculated value [35]. In this study, *COI* and *COII* genes showed an identification success of 100% for the BM criterion, while the BCM and ASB criteria showed lower identification success due to the presence of ambiguous identifications or no matches of queries in the dataset. The ambiguous identification under ASB criterion in this study was caused by a query that had only one conspecific match in the dataset (*C. bezziana* and *C. thanomthini*). In addition, “no match” under BCM and ASB criteria was caused by a query without a sequence match below the calculated threshold (*COI*: 0.72%; *COII*: 0.63%) (LC24-DL3 and LPO31-1 for *COI*, and LPO31-1 and LS33-1 for *COII*). 

In this study, the NJ tree of individual markers showed that all specimens of the same species were correctly assigned to their respective species. The results indicated that *COI* and *COII* genes are suitable for identifying Thai blow flies, even between closely related species. When examining the phylogenetic relationships reconstructed using *COI* and *COII* genes, the placement of analyzed taxa within Chrysomyinae was similar while the placement of analyzed taxa within Luciliinae was fairly different (Figure 3 and Figure 4). Within Luciliinae, the genera *Hemipyrellia*/*Hypopygiopsis* was embedded within the *Lucilia* clade based on the *COII* gene, whereas the genera *Hemipyrellia*/*Hypopygiopsis* was clearly separated from the *Lucilia* clade based on the *COI* gene. The incongruence of the tree topologies between *COI* and *COII* genes of blow flies observed in this study was not surprising because the phylogenetic placement of the blow flies often differed depending on gene of choice, gene length, species diversity, and phylogenetic tree building methods [6,8,10,11,16,37,49,50].

The present study is the first to include *H. tumrasvini*. For both *COI* and *COII*, instead of being grouped with the congeneric species *H. infumata, H. tumrasvini* was recovered as a sister to *L. cuprina* even though their adult morphology is quite different. Considering genetic distance, *L. cuprina* and *H. tumrasvini* showed interspecific distances (*COI*: 4.9%; *COII*: 4.8%) lower than the intrageneric distances observed within *Lucilia* (*COI*: 5.3–7.8%; *COII*: 5.7–9.4%), resulting in *H. tumrasvini* forming as a sister species of *L. cuprina*. The previous phylogenetic analyses of *Hypopygiopsis* demonstrated that it grouped to either *Lucilia* or *Hemipyrellia* [6,11], suggesting that its relationship with *Lucilia* and *Hemipyrellia* is ambiguous. Therefore, the use of multiple genes in different loci as well as more species of these genera is needed to resolve their phylogenetic relationships.

## 5. Conclusions

Our results demonstrated that long *COI* and *COII* sequences can be used for accurate species identification of Thai blow flies. The obtained sequences of 16 forensically important blow fly species in this study act as a reliable DNA reference database for future research on forensic entomology not only in Thailand, but also other countries where these species exist. Although this study included the majority of forensically relevant blow fly species of Thailand, most of the specimens were obtained from the northern region. Therefore, future studies should be carried out using specimens from other geographical regions of Thailand for the development of a comprehensive reference database from all geographical regions and providing additional information on the genetic diversity.

## Figures and Tables

**Table 1:** Percentage of mean intraspecific Kimura two-parameter (K2P) distances of Thai blow flies based on 1247 bp of *COI* sequences. The number of sequences, polymorphic sites, and haplotypes are shown.

Species	No. of Sequences	No. of Polymorphic Sites	No. of Haplotypes	Intraspecific Divergence (Minimum–Maximum)
*C. bezziana*	1	N/A	1	N/A
*C. megacephala*	15	8	4	0.1 (0.0–0.6)
*C. chani*	8	6	5	0.1 (0.0–0.4)
*C. pinguis*	7	14	7	0.4 (0.2–0.6)
*C. thanomthini*	2	1	2	0.1
*C. nigripes*	4	4	4	0.2 (0.1–0.2)
*C. rufifacies*	14	5	6	0.1 (0.0–0.2)
*C. villeneuvi*	14	8	6	0.1 (0.0–0.4)
*L. cuprina*	8	31	5	0.7 (0.0–2.4)
*L. papuensis*	9	12	5	0.5 (0.0–0.8)
*L. porphyrina*	6	23	6	0.7 (0.2–1.1)
*L. sinensis*	5	11	4	0.4 (0.0–0.8)
*H. ligurriens*	10	6	6	0.1 (0.0–0.4)
*H. pulchra*	4	6	4	0.3 (0.2–0.4)
*H. infumata*	3	4	3	0.2 (0.2–0.3)
*H. tumrasvini*	3	2	2	0.1 (0.0–0.2)

N/A, species represented by only one specimen; therefore, polymorphic site and intraspecific distance could not be calculated.

**Table 2:** Percentage of mean intraspecific Kimura two-parameter (K2P) distances of Thai blow flies based on 635 bp of *COII* sequences. The number of sequences, polymorphic sites, and haplotypes are shown.

Species	No. of Sequences	No. of Polymorphic Sites	No. of Haplotypes	Intraspecific Divergence (Minimum–Maximum)
*C. bezziana*	1	N/A	1	N/A
*C. megacephala*	13	0	1	0.0
*C. chani*	8	0	1	0.0
*C. pinguis*	7	3	4	0.2 (0.0–0.3)
*C. thanomthini*	2	1	2	0.2
*C. nigripes*	3	4	3	0.4 (0.3–0.6)
*C. rufifacies*	14	4	4	0.1 (0.0–0.5)
*C. villeneuvi*	14	2	3	0.1 (0.0–0.3)
*L. cuprina*	8	1	2	0.0 (0.0–0.2)
*L. papuensis*	9	5	3	0.4 (0.0–0.8)
*L. porphyrina*	6	14	5	0.8 (0.0–1.9)
*L. sinensis*	5	5	3	0.3 (0.0–0.8)
*H. pulchra*	4	2	3	0.2 (0.0–0.3)
*H. infumata*	3	1	2	0.1 (0.0–0.2)
*H. tumrasvini*	3	1	2	0.1 (0.0–0.2)

N/A, species represented by only one specimen; therefore, polymorphic site and intraspecific distance could not be calculated.

**Table 3:** Percentage of mean interspecific K2P distances of Thai blow flies based on 1247 bp of *COI* (bold) and 635 bp of *COII* (narrow) sequences.

No.	Species	Interspecific Distance															
		1	2	3	4	5	6	7	8	9	10	11	12	13	14	15	16
1	*C. bezziana*	-	3.6	5.1	3.8	3.3	5.0	6.3	6.6	9.2	10.9	9.6	11.1	-	9.4	10.0	9.4
2	*C. megacephala*	**4.9**	-	4.4	1.8	2.2	5.3	6.4	6.3	9.6	10.6	9.3	11.2	-	10.1	10.8	8.5
3	*C. chani*	**5.6**	**4.5**	-	3.9	4.8	5.3	5.8	6.5	8.9	10.0	9.7	10.7	-	7.6	8.6	7.0
4	*C. pinguis*	**5.4**	**2.6**	**4.3**	-	2.5	5.5	6.9	7.2	8.7	10.6	8.7	11.1	-	8.9	10.0	8.4
5	*C. thanomthini*	**5.6**	**3.4**	**4.5**	**2.8**	-	6.1	6.6	6.9	9.7	10.9	9.4	11.3	-	9.6	10.9	9.3
6	*C. nigripes*	**6.6**	**5.4**	**5.9**	**6.2**	**6.2**	-	6.3	6.3	10.0	11.6	10.5	11.4	-	8.4	10.1	9.1
7	*C. rufifacies*	**8.1**	**7.0**	**6.8**	**7.9**	**7.9**	**7.9**	-	3.4	9.6	10.3	9.3	10.5	-	8.4	9.7	8.3
8	*C. villeneuvi*	**7.6**	**6.7**	**7.4**	**7.8**	**7.5**	**7.7**	**4.7**	-	9.6	10.8	9.5	9.9	-	8.7	9.7	8.8
9	*L. cuprina*	**9.6**	**8.8**	**7.9**	**9.6**	**9.0**	**9.7**	**10.3**	**10.1**	-	9.4	5.7	7.0	-	5.7	6.3	4.8
10	*L. papuensis*	**10.5**	**10.7**	**8.3**	**10.7**	**10.1**	**9.9**	**10.7**	**10.0**	**6.7**	-	8.7	6.0	-	9.6	9.9	7.5
11	*L. porphyrina*	**11.1**	**10.2**	**9.2**	**10.4**	**9.8**	**11.0**	**10.7**	**10.4**	**6.2**	**7.8**	-	8.0	-	7.4	7.1	6.3
12	*L. sinensis*	**8.7**	**8.7**	**8.6**	**9.5**	**9.1**	**9.4**	**10.1**	**9.1**	**5.3**	**5.9**	**5.4**	-	-	8.9	8.9	5.8
13	*H. ligurriens*	**9.7**	**9.0**	**9.0**	**10.0**	**9.7**	**9.9**	**10.5**	**10.0**	**6.1**	**8.6**	**7.5**	**6.1**	-	-	-	-
14	*H. pulchra*	**9.3**	**9.0**	**8.4**	**9.9**	**9.4**	**9.7**	**10.2**	**9.6**	**6.1**	**8.3**	**7.2**	**5.9**	**1.1**	-	4.2	6.1
15	*H. infumata*	**9.4**	**10.2**	**9.4**	**10.0**	**10.3**	**10.0**	**11.0**	**10.3**	**7.6**	**9.0**	**9.0**	**7.1**	**5.6**	**5.5**	-	5.7
16	*H. tumrasvini*	**9.8**	**9.5**	**8.5**	**10.4**	**10.1**	**9.7**	**9.6**	**10.0**	**4.9**	**6.8**	**7.0**	**5.1**	**6.0**	**5.8**	**7.6**	-

**Table 4:** Identification success based on Best Match (BM), Best Close Match (BCM) and All Species Barcodes (ASB) criteria for *COI* (1247 bp) and *COII* (635 bp) genes.

Criteria	*COI*	*COII*
No. of sequences	114	101
No. of species	16	15
No. of sequences with closest match at 0% difference	57	80
No. of allospecific matches at 0% difference	0	0
Best Match (BM)		
- Correct identifications	100.00% (114)	100.00% (101)
- Ambiguous identifications	0.00% (0)	0.00% (0)
- Incorrect identifications	0.00% (0)	0.00% (0)
Calculated threshold for Best Close Match and All Species Barcodes	0.72%	0.63%
Best Close Match (BCM)		
- Correct identifications	98.24% (112)	98.01% (99)
- Ambiguous identifications	0.00% (0)	0.00% (0)
- Incorrect identifications	0.00% (0)	0.00% (0)
- No match closer than the calculated threshold	1.75% (2)	1.98% (2)
All Species Barcodes (ASB)		
- Correct identifications	94.73% (108)	94.05% (95)
- Ambiguous identifications	3.50% (4)	3.96% (4)
- Incorrect identifications	0.00% (0)	0.00% (0)
- No match closer than the calculated threshold	1.75% (2)	1.98% (2)

## Supplementary Materials

The following are available online at http://www.mdpi.com/2075-4450/9/4/159/s1, Figure S1: Map of Thailand showing the blow fly species collected in the sampling areas of six provinces, Table S1: Details of the Thai blow flies (Calliphoridae) and the house fly outgroup (Musidae) used in this study to obtain *COI* and *COII* sequences, Table S2: DNA polymorphism within species based on 1247 bp of *COI* sequences and their GenBank accession numbers, and Table S3: DNA polymorphism within species based on 635 bp of *COII* sequences and their GenBank accession numbers.
