# Supplementary material for: Molecular Analysis of Forensically Important Blow Flies in Thailand
Source: Insects. 2018 Nov 8;9(4):159. doi: 10.3390/insects9040159 (PMC6315464; doi:10.3390/insects9040159)
Supplement: Supplementary file 1 [file insects-09-00159-s001.pdf]

insects

MDPI

Table S1. Details of the Thai blow flies (Calliphoridae) and the house fly outgroup (Musidae) used in this study to obtain *COI* and *COII* sequences.

Species (No. of Specimens)	Voucher Code	Sex	Collection Sources and Localities	GPS Reference	Altitude (m)	Method of Preservation and Date of Collection
Chrysomya bezziana (1)	CB-D1	M	Ocular myiasis, Chiang Mai	-	-	Dried, 17 Oct 2014
Chrysomya megacephala (15)	CM0-1<br>CM0-2	M<br>M	Laboratory colony, Chiang Mai	-	-	Fresh, 22 Dec 2014
	CM2-1<br>CM2-2<br>CM2-15	M<br>M<br>M	Tham Phra Leusri, Chiang Mai	18°48' 20.252" N<br>98°54' 34.238" E	1081	85% ethanol, 26 Jun 2014
	CM25-1<br>CM25-2 *	M<br>F	Doi Khun Tan rest area, Lampang	18°23' 34.837" N<br>99°12' 54.186" E	493	85% ethanol, 8 Mar 2013
	CM26-D1	M	Khaojeak, Phatthaluang	7°36' 37.134" N<br>100°1' 58.433" E	188	Dried, 29 Apr 2013
	CM28-D1 *	M	Yantakhao, Trang	7°24' 1.032" N<br>99°40' 28.441" E	22	Dried, 1 May 2013
	CM30-1<br>CM30-2<br>CM30-45	M<br>M<br>F	Jao Dam, Nan	19°10' 45.769" N<br>100° 57' 32.63" E	309	85% ethanol, 20 Oct 2014
	CM34-1<br>CM34-2<br>CM34-20	M<br>M<br>F	Suanpa Kaokrayang, Phitsanulok	16°50' 46.732" N<br>100°44' 52.092" E	179.5	85% ethanol, 22 Oct 2014
Chrysomya chani (8)	CC21-1<br>CC21-2<br>CC21-10	M<br>M<br>F	Doi Nang Kaew, Chiang Mai	19°3' 52.991" N<br>99°22' 34.015" E	974	85% ethanol, 11 Mar 2013
	CC25-1<br>CC25-2<br>CC25-10	M<br>M<br>F	Doi Khun Tan rest area, Lampang	18°23' 34.837" N<br>99°12' 54.186" E	493	85% ethanol, 8 Mar 2013
	CC31-1<br>CC31-5	M<br>M	Ban Nalare, Nan	19°10' 47.154" N<br>100°59' 13.679" E	417	85% ethanol, 20 Oct 2014
Chrysomya pinguis (7)	CP21-1<br>CP21-2<br>CP21-3	M<br>M<br>M	Doi Nang Kaew, Chiang Mai	19°3' 52.991" N<br>99°22' 34.015" E	974	85% ethanol, 11 Mar 2013
	CP25-1<br>CP25-2	M<br>M	Doi Khun Tan rest area, Lampang	18°23' 34.837" N<br>99°12' 54.186" E	493	85% ethanol, 8 Mar 2013
	CP31-1<br>CP31-2	F<br>F	Ban Nalare, Nan	19°10' 47.154" N<br>100°59' 13.679" E	417	85% ethanol, 20 Oct 2014
Chrysomya thanomthini (2)	CT2-46	F	Tham Phra Leusri, Chiang Mai	18°48' 20.252" N	1081	85% ethanol, 9 Mar 2015

				98°54' 34.238" E		
	CT21-1	M	Doi Nang Kaew, Chiang Mai	19°3' 52.991" N<br>99°22' 34.015" E	974	85% ethanol, 11 Mar 2013
Chrysomya nigripes (4)	CN24-DF1<br>CN24-DF2 *	M<br>M	Mae Hia (forest area), Chiang Mai	18°46′01.08″ N<br>98°56′08.03″ E	344	Dried, 16 Jun 2014
	CN31-1	M	Ban Nalare, Nan	19°10' 47.154" N<br>100°59' 13.679" E	417	85% ethanol, 20 Oct 2014
	CN34-1	F	Suanpa Kaokrayang, Phitsanulok	16°50' 46.732" N<br>100°44' 52.092" E	179.5	85% ethanol, 22 Oct 2014
Chrysomya rufifacies (14)	AR0-1<br>AR0-2	M<br>M	Laboratory colony, Chiang Mai	-	-	Fresh, 12 Feb 2015
	AR24-1<br>AR24-2<br>AR24-3	M<br>M<br>M	Mae Hia (forest area), Chiang Mai	18°46′01.08″ N<br>98°56′08.03″ E	344	85% ethanol, 2 Jun 2014
	AR25-1<br>AR25-2<br>AR25-3	M<br>M<br>M	Doi Khun Tan rest area, Lampang	18°23' 34.837" N<br>99°12' 54.186" E	493	85% ethanol, 8 Mar 2013
	AR32-1<br>AR32-2<br>AR32-4	M<br>M<br>F	Ban Don Sathan, Nan	19°12' 29.293" N<br>100°56' 39.53" E	260	85% ethanol, 20 Oct 2014
	AR34-1<br>AR34-2<br>AR34-33	M<br>M<br>F	Suanpa Kaokrayang, Phitsanulok	16°50' 46.732" N<br>100°44' 52.092" E	179.5	85% ethanol, 22 Oct 2014
Chrysomya villeneuvi (14)	AV7-13<br>AV7-14<br>AV7-15	M<br>M<br>M	Sirindhorn Observatory,<br>Chiang Mai	18° 47' 21.022" N<br>98°55' 16.562" E	857	85% ethanol, 9 Mar 2015
	AV8-1<br>AV8-2	M<br>F	Sanku, Doi Suthep-Pui Mountain,<br>Chiang Mai	18°48' 56.307" N<br>98°53' 40.782" E	1595	85% ethanol, 25 Oct 2014
	AV25-1<br>AV25-2<br>AV25-3	M<br>M<br>M	Doi Khun Tan rest area, Lampang	18°23' 34.837" N<br>99°12' 54.186" E	493	85% ethanol, 8 Mar 2013
	AV30-1	M	Jao Dam, Nan	19°10' 45.769" N<br>100° 57' 32.63" E	309	85% ethanol, 20 Oct 2014
	AV31-1<br>AV31-5	M<br>F	Ban Nalare, Nan	19°10' 47.154" N<br>100°59' 13.679" E	417	85% ethanol, 20 Oct 2014
	AV34-1<br>AV34-2<br>AV34-3	F<br>F<br>F	Suanpa Kaokrayang, Phitsanulok	16°50' 46.732" N<br>100°44' 52.092" E	179.5	85% ethanol, 22 Oct 2014

Lucilia cuprina (8)	LC0-1<br>LC0-2	M<br>F	Laboratory colony, Chiang Mai	-	-	Fresh, 12 Feb 2015
	LC24-DF1<br>LC24-DF2	F<br>F	Mae Hia (forest area), Chiang Mai	18°46′01.08″ N<br>98°56′08.03″ E	344	Dried, 1 Apr 2014<br>Dried, 17 Mar 2014
	LC24-DL3	F	Mae Hia (Longan orchard),<br>Chiang Mai	18° 45′56.66″ N<br>98°55′40.13″ E	347	Dried, 4 Mar 2014
	LC34-1<br>LC34-2<br>LC34-3	M<br>M<br>M	Suanpa Kaokrayang, Phitsanulok	16°50' 46.732" N<br>100°44' 52.092" E	179.5	85% ethanol, 22 Oct 2014
Lucilia papuensis (9)	LPA2-1<br>LPA2-2	M<br>F	Tham Phra Leusri, Chiang Mai	18°48' 20.252" N<br>98°54' 34.238" E	1081	85% ethanol, 26 Jun 2014
	LPA7-19<br>LPA7-20<br>LPA7-21	M<br>M<br>M	Sirindhorn Observatory,<br>Chiang Mai	18° 47' 21.022" N<br>98°55' 16.562" E	857	85% ethanol, 9 Mar 2015
	LPA30-1	F	Jao Dam, Nan	19°10' 45.769" N<br>100° 57' 32.63" E	309	85% ethanol, 20 Oct 2014
	LPA34-1<br>LPA34-2<br>LPA34-3	M<br>M<br>F	Suanpa Kaokrayang, Phitsanulok	16°50' 46.732" N<br>100°44' 52.092" E	179.5	85% ethanol, 22 Oct 2014
Lucilia porphyrina (6)	LPO8-1<br>LPO8-2<br>LPO8-27	M<br>M<br>F	Sanku, Doi Suthep-Pui Mountain,<br>Chiang Mai	18°48' 56.307" N<br>98°53' 40.782" E	1595	85% ethanol, 25 Oct 2014
	LPO31-1<br>LPO31-2<br>LPO31-7	M<br>M<br>F	Ban Nalare, Nan	19°10' 47.154" N<br>100°59' 13.679" E	417	85% ethanol, 20 Oct 2014
Lucilia sinensis (5)	LS31-1	F	Ban Nalare, Nan	19°10' 47.154" N<br>100°59' 13.679" E	417	85% ethanol, 20 Oct 2014
	LS33-1	F	Huanam mushroom farm, Nan	19° 8' 12.536" N<br>100°57' 23.327" E	369	85% ethanol, 21 Oct 2014
	LS37-1<br>LS37-2<br>LS37-3	M<br>M<br>M	Huay Toeng Thao, Chiang Mai	18°52' 1.064" N<br>98°54' 12.281" E	762.5	85% ethanol, 17 Oct 2014
Hemipyrellia ligurriens (10)	HL24-DP29<br>HL24-DP31	M<br>M	Mae Hia (palm plantation),<br>Chiang Mai	18°45′27.841″ N<br>98°55′48.515″ E	330	Dried, 12 Sep 2013
	HL28-D1<br>HL28-D2	M<br>M	Yantakhao, Trang	7°24' 1.032" N<br>99°40' 28.441" E	22	Dried, 1 May 2013
	HL30-1<br>HL30-2	M<br>M	Jao Dam, Nan	19°10' 45.769" N<br>100° 57' 32.63" E	309	85% ethanol, 20 Oct 2014

	HL30-3	M				
	HL34-1<br>HL34-2<br>HL34-3	M<br>M<br>M	Suanpa Kaokrayang, Phitsanulok	16°50' 46.732" N<br>100°44' 52.092" E	179.5	85% ethanol, 22 Oct 2014
Hemipyrellia pulchra (4)	HP24-DL1<br>HP24-DL2	F<br>F	Mae Hia (Longan orchard), Chiang Mai	18° 45′56.66″ N<br>98°55′40.13″ E	347	Dried, 2 Jun 2014
	HP34-1	F	Suanpa Kaokrayang, Phitsanulok	16°50' 46.732" N<br>100°44' 52.092" E	179.5	85% ethanol, 22 Oct 2014
	HP36-1	F	Huay Nam Fong, Phitsanulok	16° 51' 8.408" N<br>100°31' 4.912" E	122.5	85% ethanol, 22 Oct 2014
Hypopygiopsis infumata (3)	HI7-22	M	Sirindhorn Observatory, Chiang Mai	18° 47' 21.022" N<br>98°55' 16.562" E	857	85% ethanol, 9 Mar 2015
	HI30-1<br>HI30-2	M<br>F	Jao Dam, Nan	19°10' 45.769" N<br>100° 57' 32.63" E	309	85% ethanol, 20 Oct 2014
Hypopygiopsi tumrasavini (3)	HT2-1	F	Tham Phra Leusri, Chiang Mai	18°48' 20.252" N<br>98°54' 34.238" E	1081	85% ethanol, 31 Jul 2014
	HT8-1	F	Sanku, Doi Suthep-Pui Mountain, Chiang Mai	18°48' 56.307" N<br>98°53' 40.782" E	1595	85% ethanol, 25 Oct 2014
	HT31-1	F	Ban Nalare, Nan	19°10' 47.154" N<br>100°59' 13.679" E	417	85% ethanol, 20 Oct 2014
Musca domestica (2)	MD0-1<br>MD0-2	F<br>M	Laboratory colony, Chiang Mai	-	-	Fresh, 16 Mar 2015

* Samples of which only *COI* data were obtained.

Table S2. DNA polymorphism within species based on 1247 bp of *COI* sequences and their GenBank accession numbers.

Species (No. of Sequences)	No. of Polymorphic Sites (No. of Haplotypes)	Haplotype (No. of Each Haplotype)	Voucher Code of Specimens in Each Haplotype	Province	Representative Specimens of Each Haplotype	GenBank Accession No.
Chrysomya bezziana (1)	N/A (1)	Haplotype 1 (1)	CB-D1	Chiang Mai	CB-D1	KR921597
Chrysomya megacephala (15)	8 (4)	Haplotype 1 (12)	CM0-1, CM0-2	Lab colony	CM0-1	KR921598
			CM2-1, CM2-2, CM2-15	Chiang Mai	CM2-1	KR921599
			CM25-1, CM25-2	Lampang	CM25-1	KR921600
			CM30-1, CM30-2, CM30-45	Nan	CM30-1	KR921603
			CM34-1, CM34-20	Phitsanulok	CM34-1	KR921604
		Haplotype 2 (1)	CM26-D1	Phatthaluang	CM26-D1	KR921601
		Haplotype 3 (1)	CM28-D1	Trang	CM28-D1	KR921602
		Haplotype 4 (1)	CM34-2	Phitsanulok	CM34-2	KR921605
Chrysomya chani (8)	6 (5)	Haplotype 1 (4)	CC21-1	Chiang Mai	CC21-1	KR921606
			CC25-1, CC25-2, CC25-10	Lampang	CC25-1	KR921609
		Haplotype 2 (1)	CC21-2	Chiang Mai	CC21-2	KR921607
		Haplotype 3 (1)	CC21-10	Chiang Mai	CC21-10	KR921608
		Haplotype 4 (1)	CC31-1	Nan	CC31-1	KR921610
		Haplotype 5 (1)	CC31-5	Nan	CC31-5	KR921611
Chrysomya pinguis (7)	14 (7)	Haplotype 1 (1)	CP21-1	Chiang Mai	CP21-1	KR921612
		Haplotype 2 (1)	CP21-2	Chiang Mai	CP21-2	KR921613
		Haplotype 3 (1)	CP21-3	Chiang Mai	CP21-3	KR921614
		Haplotype 4 (1)	CP25-1	Lampang	CP25-1	KR921615
		Haplotype 5 (1)	CP25-2	Lampang	CP25-2	KR921616
		Haplotype 6 (1)	CP31-1	Nan	CP31-1	KR921617
		Haplotype 7 (1)	CP31-2	Nan	CP31-2	KR921618
Chrysomya thanomthini (2)	1 (2)	Haplotype 1 (1)	CT2-46	Chiang Mai	CT2-46	KR921619
		Haplotype 2 (1)	CT21-1	Chiang Mai	CT21-1	KR921620
Chrysomya nigripes (4)	4 (4)	Haplotype 1 (1)	CN24-DF1	Chiang Mai	CN24-DF1	KR921621
		Haplotype 2 (1)	CN24-DF2	Chiang Mai	CN24-DF2	KR921622
		Haplotype 3 (1)	CN31-1	Nan	CN31-1	KR921623
		Haplotype 4 (1)	CN34-1	Phitsanulok	CN34-1	KR921624
Chrysomya rufifacies (14)	5 (6)	Haplotype 1 (2)	AR0-1, AR0-2	Lab colony	AR0-1	KR921625

		Haplotype 2 (8)	AR24-1, AR24-2	Chiang Mai	AR24-1	KR921627
			AR25-1, AR25-2, AR25-3	Lampang	AR25-1	KR921628
			AR32-2	Nan	AR32-2	KR921630
			AR34-2, AR34-33	Phitsanulok	AR34-2	KR921633
		Haplotype 3 (1)	AR24-3	Chiang Mai	AR24-3	KR921626
		Haplotype 4 (1)	AR32-1	Nan	AR32-1	KR921629
		Haplotype 5 (1)	AR32-4	Nan	AR32-4	KR921631
		Haplotype 6 (1)	AR34-1	Phitsanulok	AR34-1	KR921632
Chrysomya villeneuvi (14)	8 (6)	Haplotype 1 (1)	AV7-13	Chiang Mai	AV7-13	KR921634
		Haplotype 2 (1)	AV7-14	Chiang Mai	AV7-14	KR921635
		Haplotype 3 (9)	AV7-15	Chiang Mai	AV7-15	KR921636
			AV25-1, AV25-2	Lampang	AV25-1	KR921639
			AV30-1	Nan	AV30-1	KR921641
			AV31-1, AV31-5	Nan	AV31-1	KR921642
			AV34-1, AV34-2, AV34-3	Phitsanulok	AV34-1	KR921643
		Haplotype 4 (1)	AV8-1	Chiang Mai	AV8-1	KR921637
		Haplotype 5 (1)	AV8-2	Chiang Mai	AV8-2	KR921638
		Haplotype 6 (1)	AV25-3	Lampang	AV25-3	KR921640
Lucilia cuprina (8)	31 (5)	Haplotype 1 (4)	LC0-1, LC0-2	Lab colony	LC0-1	KR921644
			LC24-DF1	Chiang Mai	LC24-DF1	KR921645
			LC34-1	Phitsanulok	LC34-1	KR921648
		Haplotype 2 (1)	LC24-DF2	Chiang Mai	LC24-DF2	KR921646
		Haplotype 3 (1)	LC24-DL3	Chiang Mai	LC24-DL3	KR921647
		Haplotype 4 (1)	LC34-2	Phitsanulok	LC34-2	KR921649
		Haplotype 5 (1)	LC34-3	Phitsanulok	LC34-3	KR921650
Lucilia papuensis (9)	12 (5)	Haplotype 1 (3)	LPA2-1, LPA2-2	Chiang Mai	LPA2-1	KR921651
			LPA7-21	Chiang Mai	LPA7-21	KR921654
		Haplotype 2 (1)	LPA7-19	Chiang Mai	LPA7-19	KR921652
		Haplotype 3 (1)	LPA7-20	Chiang Mai	LPA7-20	KR921653
		Haplotype 4 (2)	LPA30-1	Nan	LPA30-1	KR921655
			LPA34-2	Phitsanulok	LPA34-2	KR921657
		Haplotype 5 (2)	LPA34-1, LPA34-3	Phitsanulok	LPA34-1	KR921656
Lucilia porphyrina (6)	23 (6)	Haplotype 1 (1)	LPO8-1	Chiang Mai	LPO8-1	KR921658
		Haplotype 2 (1)	LPO8-2	Chiang Mai	LPO8-2	KR921659

		Haplotype 3 (1)	LPO8-27	Chiang Mai	LPO8-27	KR921660
		Haplotype 4 (1)	LPO31-1	Nan	LPO31-1	KR921661
		Haplotype 5 (1)	LPO31-2	Nan	LPO31-2	KR921662
		Haplotype 6 (1)	LPO31-7	Nan	LPO31-7	KR921663
Lucilia sinensis (5)	11 (4)	Haplotype 1 (2)	LS31-1	Nan	LS31-1	KR921664
			LS37-1	Chiang Mai	LS37-1	KR921666
		Haplotype 2 (1)	LS33-1	Nan	LS33-1	KR921665
		Haplotype 3 (1)	LS37-2	Chiang Mai	LS37-2	KR921667
		Haplotype 4 (1)	LS37-3	Chiang Mai	LS37-3	KR921668
Hemipyrellia ligurriens (10)	6 (6)	Haplotype 1 (5)	HL24-DP29, HL24-DP31	Chiang Mai	HL24-DP29	KR921669
			HL30-1, HL30-2	Nan	HL30-1	KR921672
			HL34-1	Phitsanulok	HL34-1	KR921674
		Haplotype 2 (1)	HL28-D1	Trang	HL28-D1	KR921670
		Haplotype 3 (1)	HL28-D2	Trang	HL28-D2	KR921671
		Haplotype 4 (1)	HL30-3	Nan	HL30-3	KR921673
		Haplotype 5 (1)	HL34-2	Phitsanulok	HL34-2	KR921675
		Haplotype 6 (1)	HL34-3	Phitsanulok	HL34-3	KR921676
Hemipyrellia pulchra (4)	6 (4)	Haplotype 1 (1)	HP24-DL1	Chiang Mai	HP24-DL1	KR921677
		Haplotype 2 (1)	HP24-DL2	Chiang Mai	HP24-DL2	KR921678
		Haplotype 3 (1)	HP34-1	Phitsanulok	HP34-1	KR921679
		Haplotype 4 (1)	HP36-1	Phitsanulok	HP36-1	KR921680
Hypopygiopsis infumata (3)	4 (3)	Haplotype 1 (1)	HI7-22	Chiang Mai	HI7-22	KR921681
		Haplotype 2 (1)	HI30-1	Nan	HI30-1	KR921682
		Haplotype 3 (1)	HI30-2	Nan	HI30-2	KR921683
Hypopygiopsis tumrasvini (3)	2 (2)	Haplotype 1 (2)	HT2-1	Chiang Mai	HT2-1	KR921684
			HT8-1	Chiang Mai	HT8-1	KR921685
		Haplotype 2 (1)	HT31-1	Nan	HT31-1	KR921686
Musca domestica (2)	9 (2)	Haplotype 1 (1)	MD0-1	Lab colony	MD0-1	KR921687
		Haplotype 2 (1)	MD0-2	Lab colony	MD0-2	KR921688

N/A, species represented by only one specimen; therefore, polymorphic site could not be calculated.

Table S3. DNA polymorphism within species based on 635 bp of *COII* sequences and their GenBank accession numbers.

Species (No. of Sequences)	No. of Polymorphic Sites (No. of Haplotypes)	Haplotype (No. of Each Haplotype)	Voucher Code of Specimens in Each Haplotype	Province	Representative Specimen of Each Haplotype	GenBank Accession No.
Chrysomya bezziana (1)	N/A (1)	Haplotype 1 (1)	CB-D1	Chiang Mai	CB-D1	KU556169
Chrysomya megacephala (13)	0 (1)	Haplotype 1 (13)	CM0-1, CM0-2	Lab colony	CM0-1	KU556170
			CM2-1, CM2-2, CM2-15	Chiang Mai	CM2-1	KU556171
			CM25-1	Lampang	CM25-1	KU556172
			CM26-D1	Phatthaluang	CM26-D1	KU556173
			CM30-1, CM30-2, CM30-15	Nan	CM30-1	KU556174
			CM34-1, CM34-2, CM34-20	Phitsanulok	CM34-1	KU556175
Chrysomya chani (8)	0 (1)	Haplotype 1 (8)	CC21-1, CC21-2, CC21-10	Chiang Mai	CC21-1	KU556176
			CC25-1, CC25-2, CC25-10	Lampang	CC25-1	KU556177
			CC31-1, CC31-5	Nan	CC31-1	KU556178
Chrysomya pinguis (7)	3 (4)	Haplotype 1 (3)	CP21-1, CP21-3	Chiang Mai	CP21-1	KU556179
			CP25-1	Lampang	CP25-1	KU556181
		Haplotype 2 (1)	CP21-2	Chiang Mai	CP21-2	KU556180
		Haplotype 3 (2)	CP25-2	Lampang	CP25-2	KU556182
			CP31-1	Nan	CP31-1	KU556183
		Haplotype 4 (1)	CP31-2	Nan	CP31-2	KU556184
Chrysomya thanomthini (2)	1 (2)	Haplotype 1 (1)	CT2-46	Chiang Mai	CT2-46	KU556185
		Haplotype 2 (1)	CT21-1	Chiang Mai	CT21-1	KU556186
Chrysomya nigripes (3)	4 (3)	Haplotype 1 (1)	CN24-DF1	Chiang Mai	CN24-DF1	KU556187
		Haplotype 2 (1)	CN31-1	Nan	CN31-1	KU556188
		Haplotype 3 (1)	CN34-1	Phitsanulok	CN34-1	KU556189
Chrysomya rufifacies (14)	4 (4)	Haplotype 1 (2)	AR0-1, AR0-2	Lab colony	AR0-1	KU556190
		Haplotype 2 (8)	AR24-1, AR24-2, AR24-3	Chiang Mai	AR24-1	KU556191
			AR25-2, AR25-3	Lampang	AR25-2	KU556193
			AR32-4	Nan	AR32-4	KU556196
			AR34-1, AR34-2	Phitsanulok	AR34-1	KU556197
		Haplotype 3 (3)	AR25-1	Lampang	AR25-1	KU556192
			AR32-1	Nan	AR32-1	KU556194
			AR34-33	Phitsanulok	AR34-33	KU556198

		Haplotype 4 (1)	AR32-2	Nan	AR32-2	KU556195
Chrysomya villeneuvi (14)	2 (3)	Haplotype 1 (12)	AV7-13, AV7-14, AV7-15	Chiang Mai	AV7-13	KU556199
			AV8-2	Chiang Mai	AV8-2	KU556201
			AV25-1, AV25-2, AV25-3	Lampang	AV25-1	KU556202
			AV30-1	Nan	AV30-1	KU556203
			AV31-1, AV31-5	Nan	AV31-1	KU556204
			AV34-1, AV34-3	Phitsanulok	AV34-1	KU556205
		Haplotype 2 (1)	AV8-1	Chiang Mai	AV8-1	KU556200
		Haplotype 3 (1)	AV34-2	Phitsanulok	AV34-2	KU556206
Lucilia cuprina (8)	1 (2)	Haplotype 1 (7)	LC0-1, LC0-2	Lab colony	LC0-1	KU556207
			LC24-DF1, LC24-DF2	Chiang Mai	LC24-DF1	KU556208
			LC34-1, LC34-2, LC34-3	Phitsanulok	LC34-1	KU556210
		Haplotype 2 (1)	LC24-DL3	Chiang Mai	LC24-DL3	KU556209
Lucilia papuensis (9)	5 (3)	Haplotype 1 (4)	LPA2-1, LPA2-2	Chiang Mai	LPA2-1	KU556211
			LPA7-20, LPA7-21	Chiang Mai	LPA7-20	KU556213
		Haplotype 2 (3)	LPA7-19	Chiang Mai	LPA7-19	KU556212
			LPA34-1, LPA34-3	Phitsanulok	LPA34-1	KU556215
		Haplotype 3 (2)	LPA30-1	Nan	LPA30-1	KU556214
			LPA34-2	Phitsanulok	LPA34-2	KU556216
Lucilia porphyrina (6)	14 (5)	Haplotype 1 (1)	LPO8-1	Chiang Mai	LPO8-1	KU556217
		Haplotype 2 (1)	LPO8-2	Chiang Mai	LPO8-2	KU556218
		Haplotype 3 (1)	LPO8-27	Chiang Mai	LPO8-27	KU556219
		Haplotype 4 (1)	LPO31-1	Nan	LPO31-1	KU556220
		Haplotype 5 (2)	LPO31-2, LPO31-7	Nan	LPO31-2	KU556221
Lucilia sinensis (5)	5 (3)	Haplotype 1 (3)	LS31-1	Nan	LS31-1	KU556222
			LS37-1, LS37-2	Chiang Mai	LS37-1	KU556224
		Haplotype 2 (1)	LS33-1	Nan	LS33-1	KU556223
		Haplotype 3 (1)	LS37-3	Chiang Mai	LS37-3	KU556225
Hemipyrellia pulchra (4)	2 (3)	Haplotype 1 (2)	HP24-DL1	Chiang Mai	HP24-DL1	KU556226
			HP34-1	Phitsanulok	HP34-1	KU556228
		Haplotype 2 (1)	HP24-DL2	Chiang Mai	HP24-DL2	KU556227
		Haplotype 3 (1)	HP36-1	Phitsanulok	HP36-1	KU556229
Hypopygiopsis infumata (3)	1 (2)	Haplotype 1 (2)	HI7-22	Chiang Mai	HI7-22	KU556230

			HI30-1	Nan	HI30-1	KU556231
		Haplotype 2 (1)	HI30-2	Nan	HI30-2	KU556232
Hypopygiopsis tumrasvini (3)	1 (2)	Haplotype 1 (1)	HT2-1	Chiang Mai	HT2-1	KU556233
		Haplotype 2 (2)	HT8-1	Chiang Mai	HT8-1	KU556234
			HT31-1	Nan	HT31-1	KU556235
Musca domestica (2)	0 (1)	Haplotype 1 (2)	MD0-1, MD0-2	Lab colony	MD0-1	KU556236

N/A, species represented by only one specimen; therefore, polymorphic site could not be calculated.

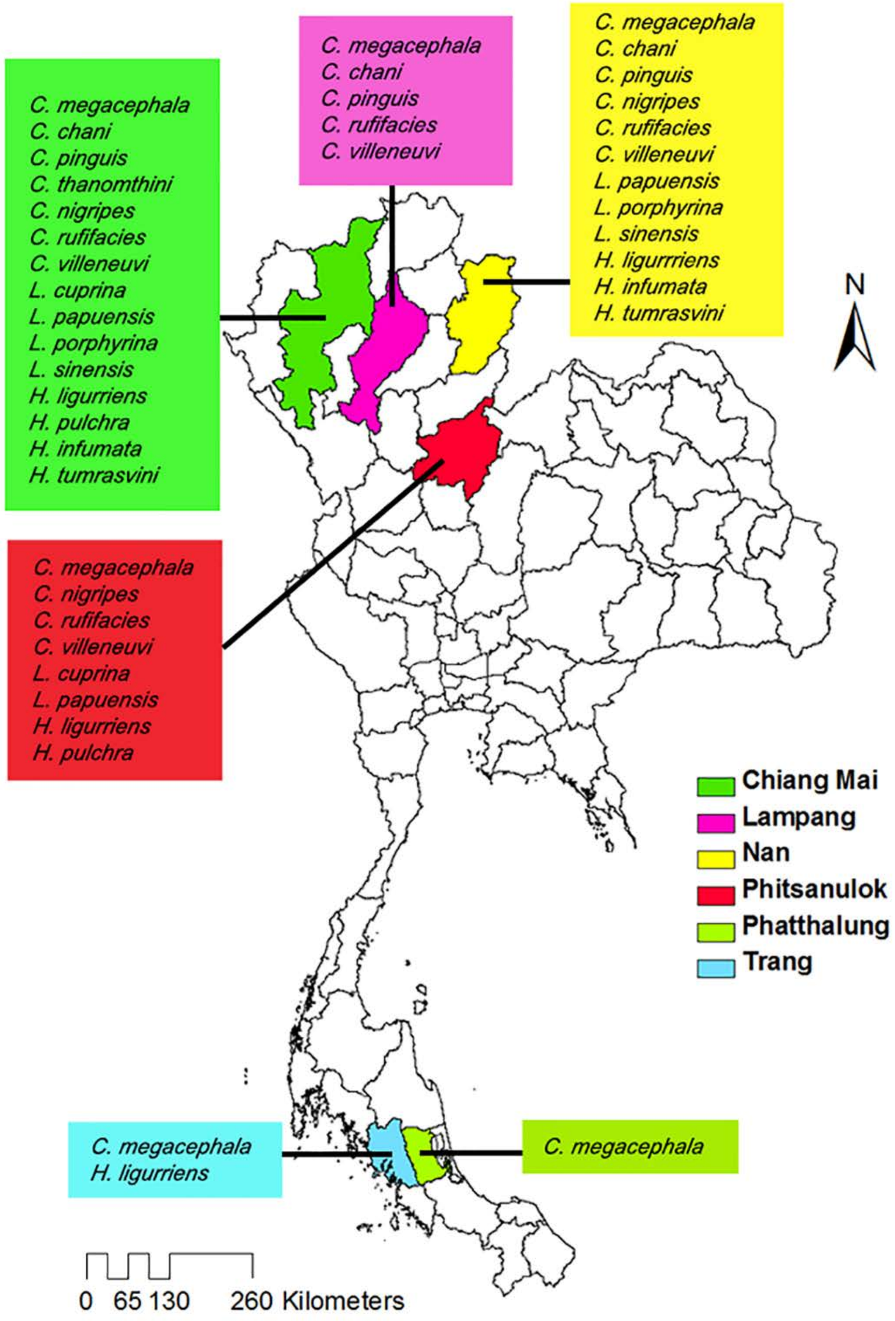

Figure S1. Map of Thailand showing the blow fly species collected in the sampling areas of six provinces.
